# Adrenal Hypoplasia: A Diagnostic and Clinical Challenge

**DOI:** 10.7759/cureus.78074

**Published:** 2025-01-27

**Authors:** Sara Geraldes Paulino, Alice Porto Guerra Vasconcelos, Sofia Ferreira, Carla Costa, Rita Santos Silva, Cintia Castro-Correia

**Affiliations:** 1 Pediatrics, Centro Hospitalar Universitário de São João, Porto, PRT; 2 Genetics, Centro Hospitalar Universitário de São João, Porto, PRT; 3 Pediatrics, Centro Hospitalar Universitário de São João, Porto, PRT

**Keywords:** adrenal hypoplasia congenita, adrenal insufficiency, genetic syndromes, hypogonadotropic hypogonadism, salt-wasting crisis

## Abstract

Adrenal insufficiency can be life-threatening and results from inadequate secretion of hormones by the adrenal cortex. In pediatric patients, the most common cause is congenital adrenal hyperplasia due to 21-hydroxylase deficiency. We present a clinical case of a 17-year-old male. During the neonatal period, he experienced a salt-wasting crisis with shock, hyponatremia, metabolic acidosis, and elevated adrenocorticotropic hormone (ACTH) levels, with normal 17-hydroxyprogesterone. Hydrocortisone and fludrocortisone were initiated. Ten months later, genetic testing for the *CYP21A2* gene was normal, and 17-hydroxyprogesterone levels were low, prompting the tapering of medication. However, another salt-wasting crisis led to the resumption of treatment. The patient presented a clear need for high doses of glucocorticoids to maintain symptomatic control. Genetic testing revealed a deletion in the *CSNK2A1* gene, linked to Okur-Chung syndrome, along with a pathogenic *NR0B1* variant, confirming adrenal hypoplasia congenita and hypogonadotropic hypogonadism. Hormone replacement and testosterone supplementation improved growth and pubertal development. The case improves our understanding of the phenotypic range and diagnostic challenges associated with NR0B1-related adrenal hypoplasia. Besides, the concurrent diagnosis of a second genetic disorder created additional challenges for both diagnoses. It emphasizes the importance of comprehensive clinical, biochemical, and genetic assessment to avoid misdiagnosis and ensure appropriate management strategies for complex endocrine disorders.

## Introduction

Adrenal insufficiency (AI) is a potentially life-threatening condition characterized by inadequate secretion of glucocorticoid and/or mineralocorticoid hormones by the adrenal cortex [1]. Neonatal adrenal crisis arises from congenital AI and it can manifest within the first two weeks of life and is characterized by hyponatremia, hyperkalemia, hypotension, and, occasionally, hypoglycemia [2]. The most common cause of AI in pediatric patients is congenital adrenal hyperplasia (CAH), which is an autosomal recessive hereditary disorder caused by deficiencies in various enzymes (StAR, 3-βHSD, 17α-OH, 21-OH, and 11β-OH) [3]. CAH due to 21-hydroxylase deficiency accounts for about 95% of cases in the pediatric population. To reduce morbidity and mortality, immediate recognition and management of adrenal crisis are essential, especially since this condition is not included in neonatal screening in Portugal [1,4].

## Case presentation

We report the case of a 17-year-old male adolescent with AI. Informed consent was obtained from the patient’s mother for both the genetic investigation and the publication of the clinical case. He was born at 40 weeks of gestation after an uncomplicated pregnancy. The father had short stature and learning difficulties, but the remaining family history was unremarkable. At birth, he presented with signs of respiratory distress, cyanosis, and hypoxemia, leading to admission to the Neonatal Intensive Care Unit, with suspected neonatal sepsis, to initiate non-invasive mechanical ventilation. Physical examination showed no alterations, except for hyperpigmentation of the scrotum. On the third day of life, he developed refractory hypotension to aminergic support and laboratory tests showed hyponatremia, hyperkalemia, and metabolic acidosis - suspected of salt-wasting adrenal crisis. The analytical study showed low levels of cortisol and aldosterone and high levels of adrenocorticotropic hormone (ACTH), 17-hydroxyprogesterone (17-OHP), dehydroepiandrosterone-sulfate (DHEA-S), androstenedione and testosterone. Therefore, he was started on hydrocortisone and fludrocortisone for suspected CAH and was discharged on the 30th day of life. At 10 months of age, as the levels of 17 OHP were low (1.2 ng/dL), and targeted sequencing for the nine most common European variants in the *CYP21A2* gene was performed and did not reveal any of these variants. The CAH hypothesis was questioned and medication tapering was initiated. However, at 22 months of age, hyperpigmentation of the skin and mucous membranes began to appear. Laboratory tests showed low levels of aldosterone (7.3 ng/dL) and elevated renin, as well as low levels of DHEA-S (4.7 ng/mL), androstenedione (<0.3 ng/mL) and testosterone (<0.08 ng/mL) and high levels of ACTH. At this time, the patient presented another salt-wasting crisis and restarted glucocorticoid treatment. Subsequently, it became evident that the patient required high doses of glucocorticoids (20 mg/m²/day of hydrocortisone) to maintain symptomatic control, as lower doses were associated with hypoglycemia, asthenia, vomiting, and hyperpigmentation of the skin and mucous membranes, along with markedly elevated ACTH levels (>2000 pg/mL) (Table 1).

**Table 1:** Hormonal values ACTH: adrenocorticotropic hormone; 17-OHP: 17-hydroxyprogesterone; DHEA-S: dehydroepiandrosterone sulfate

Normal hormonal values			Patient’s values at 22 months old
Cortisol (µg/dL)	1-5 years	6-25	24.5
ACTH (pg/mL)	6 months to 4 years	2.5-54.3	>2000
17-OHP (ng/dL)	1-5 years	4-114	<0.1
DHEA-S (ng/mL)	1-5 years	8-141	4.7
Androstenedione (ng/dL)	1-5 years	5-51	<0.3
Testosterone (ng/mL)	7-12 months, Tanner I	≤0.16, 0.02-0.23	<0.08

As part of the etiological investigation, a 250 µg ACTH stimulation test (Synacthen®; Atnahs Pharma UK Ltd., Basildon, UK) confirmed primary AI, with cortisol levels measured at 44 µg/dL at baseline (0 minutes) and 18.2 µg/dL at 60 minutes; an immunological study was conducted (thyroid antibodies, adrenal antibodies, antinuclear antibodies (ANA), antineutrophil cytoplasmic antibodies (ANCA)) and showed no abnormalities; and measurement of very long-chain fatty acids was also carried out, excluding X-linked adrenoleukodystrophy. Additionally, a full *CYP21A2*gene sequencing and copy number variation screening were performed, along with a multigene panel associated with CAH, and no variations associated with this pathology were identified. Thus, the etiology of primary AI remained unknown.

**Figure 1:**
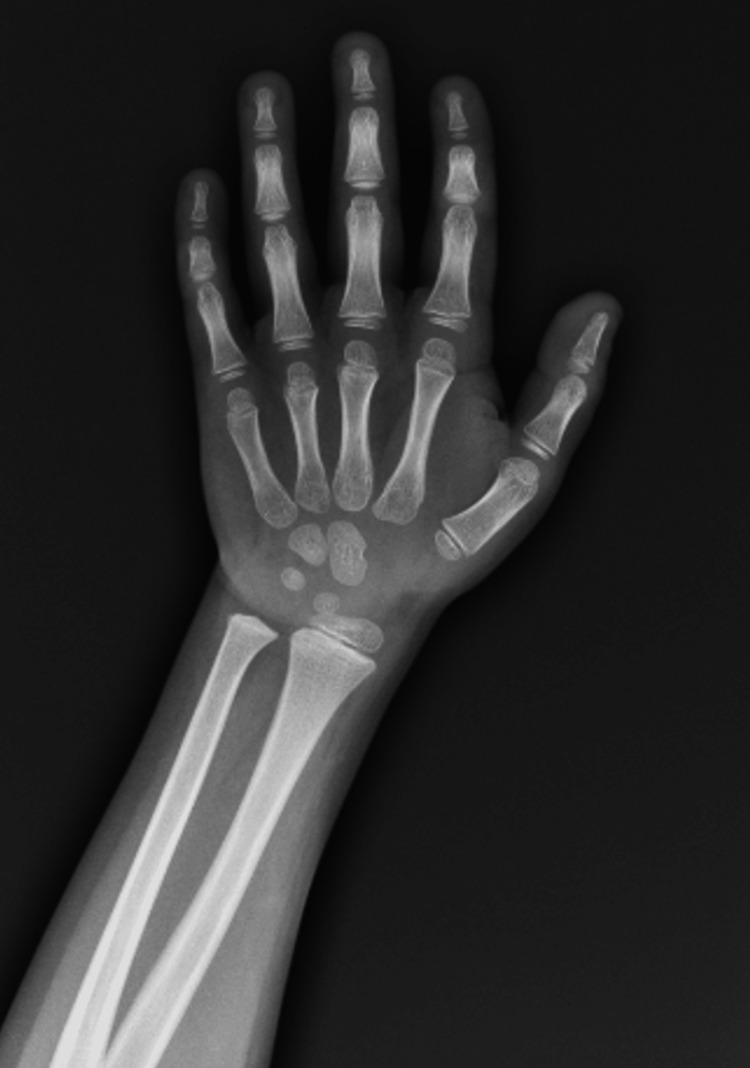
Bone age at 17 years old (left hand/wrist). The patient’s chronological age was 17 years, while his bone age was 15 years, showing a delay of two years.

Besides the AI, throughout his childhood and adolescence, he presented a short stature (percentile <1 on the WHO growth charts), a pubertal delay with hypogonadotropic hypogonadism, and delayed bone age - delay of two years relative to chronological age (Figure 1). Initially, these issues were attributed to high doses of corticosteroids used for treatment. However, he consistently required high doses of hydrocortisone and fludrocortisone, experiencing decompensation each time attempts were made to reduce medication, presenting with asthenia, hyperpigmentation in skin folds, scrotum, and gums. Moreover, he presented a broad nasal bridge, upturned nose, and epicanthal folds and was also diagnosed with a mild intellectual developmental disorder, based on formal cognitive assessment.

At age 15, a medical geneticist was involved and a genomic array test was conducted, identifying a heterozygous deletion at cytoband 20p13 encompassing the entire *CSNK2A1* gene, known to be associated with Okur-Chung syndrome. This deletion was inherited from the father and presumably leads to haploinsufficiency, possibly explaining the short stature and intellectual disability observed, though it does not account for the AI.

Hence, a multigene next-generation sequencing (NGS) panel for genes associated with congenital adrenal hypoplasia (*CDKN1C, NNT, NR0B1, POLE, POMC, POR, SAMD9 e TBX19*) was conducted and the pathogenic variant in the *NR0B1 gene* c.1273A>G p.(Arg425Gly) was found. Maternal testing confirmed that this variant is *de novo* and is responsible not only for the AI but also for the hypogonadotropic hypogonadism. The patient began testosterone treatment, resulting in a positive development in the pubertal stage, progressing from Tanner stage 1 to Tanner stage 2 within six months. Additionally, the growth rate increased from 2 cm/year (P<3) to 8 cm/year (P>97). He is now 17 years old. He is being treated with hydrocortisone 17.7 mg/m²/day and fludrocortisone 100 mcg/day, has 152 cm (<P5), 72.25 kg (P 75-90), Tanner stage 2 and presents no symptoms of AI.

## Discussion

This case highlights the diagnostic complexities and clinical variability of adrenal hypoplasia congenita, particularly in the context of comorbid genetic conditions. The patient presented with a salt-wasting adrenal crisis at birth, initially raising suspicion for CAH. This common initial misdiagnosis arises due to the overlapping electrolyte imbalances and hormonal profiles that both conditions can exhibit in early life [2,4]. However, the low 17 OH-progesterone levels at 10 months of age, along with the subsequent hormonal profile showing low DHEA-S, androstenedione, and testosterone, were inconsistent with classic CAH [2,5]. Genetic testing plays a critical role in distinguishing adrenal hypoplasia from other causes of AI [6]. The identification of an *NR0B1 *gene variant (c. 1273 A>G (p. (Arg425Gly))) confirmed the diagnosis of adrenal hypoplasia congenita [7]. X-linked adrenal hypoplasia congenita (OMIM # 300200) is a rare disorder, caused by deletions or single nucleotide variants in the *NR0B1 (DAX1) *gene. Its incidence remains undetermined and mutations in this gene are known to cause a spectrum of phenotypes, ranging from isolated AI to combined AI and hypogonadotropic hypogonadism, as seen in this patient [8]. Patients with *NR0B1 *variants typically present in early infancy, often within the first few weeks of life, with symptoms of AI such as lethargy, vomiting, dehydration, and failure to thrive, however, they can have a more insidious onset during childhood [6,8].

The salt-wasting form, as seen in this case, is the most common presentation, characterized by hyponatremia, hyperkalemia, and metabolic acidosis [8-10]. Hypoglycemia is not always present, as reported in a case study of a boy with a deletion of the entire *NR0B1 *gene [8]. Laboratory investigations often reveal low levels of cortisol and aldosterone, elevated levels of renin (due to lack of aldosterone feedback on the renin-angiotensin system), and increased ACTH levels [9,10]. Hypogonadotropic hypogonadism, often emerging later in childhood or adolescence, manifests with delayed puberty, underdeveloped secondary sexual characteristics, and infertility, as observed in the case of an adolescent who, at 17 years old, presented with an absence of secondary sexual characteristics [6,11]. This patient also presented with short stature and mild intellectual developmental disorder, at first interpreted in the context of treatment with high doses of hydrocortisone, but later attributed to a deletion encompassing the *CSNK2A1 *gene and consequent diagnosis of Okur-Chung syndrome (OMIM # 617062) [12]. Okur-Chung syndrome is a rare genetic condition characterized by developmental delay, intellectual disability, and distinctive facial features, such as those outlined above, observed in this patient [13,14]. The severity of these features can vary significantly, ranging from mild learning difficulties to more profound intellectual disabilities [12,13]. Diagnosis is based on clinical findings and confirmed by genetic testing, which typically identifies a loss of function variant in the *CSNK2A1 *gene [12,15]. This case underscores the importance of considering multiple genetic diagnoses in patients with complex clinical presentations. While the *NR0B1 *variant accounts for AI and hypogonadotropic hypogonadism, the Okur-Chung syndrome contributes to the overall growth and developmental profile.

## Conclusions

Adrenal hypoplasia congenita should be included in the differential diagnosis of any infant presenting with AI, even if the initial presentation closely resembles CAH. In fact, genetic testing is essential for the definitive diagnosis of adrenal hypoplasia congenita and to distinguish it from other etiologies of AI. The coexistence of other rare genetic conditions can further complicate the phenotype of patients with adrenal hypoplasia congenita, highlighting the importance of comprehensive genetic evaluation in such complex cases. In conclusion, this case report contributes to the understanding of the phenotypic spectrum and diagnostic challenges associated with *NR0B1* variations. It also emphasizes the importance of looking at the patient as a whole, integrating clinical, biochemical, and genetic findings, in order to avoid false or misdiagnosis and to ensure appropriate management strategies in patients with complex endocrine disorders.
